# When to call it off: Defining the role of low-dose corticosteroids in thwarting the progression of non-severe COVID-19

**DOI:** 10.1016/j.amsu.2021.01.063

**Published:** 2021-01-21

**Authors:** Talal Almas, Salman Hussain, Abdul Haadi, Maryam Ehtesham, Afnan Hassan, Abdul Wali Khan, Aamir Hameed

**Affiliations:** a RCSI University of Medicine and Health Sciences, Dublin, Ireland; b Simon Fraser University, Surrey, British Columbia, Canada; c College of Physicians and Surgeons, Pakistan; d Tissue Engineering Research Group (TERG), Department of Anatomy and Regenerative Medicine, RCSI University of Medicine and Health Sciences, Dublin, Ireland

The coronavirus disease 2019 (COVID-19) continues to wreak havoc, culminating in soaring morbidity and mortality worldwide. To date, a plethora of pharmaceutical regimens has been employed in the treatment of the infection, but management of the ailment still remains largely supportive. In addition to antivirals such as remdesivir, azithromycin and tocilizumab also remain the cornerstone of current therapeutic management. Recent research has divulged that the exorbitantly high fatality and morbidity rates associated with COVID-19 can be attributed, in part, to an aberrant and dysregulated immune response that ensues secondary to the cytokine storm observed in COVID-19 [1]. Corticosteroids have traditionally remained exceedingly imperative in the management of hyperinflammatory states. Owing to their potent anti-inflammatory properties, corticosteroids have remained at the epicentre of a therapeutic conundrum surrounding the optimal management of COVID-19. To this end, the RECOVERY trial yielded conspicuous evidence that the administration of dexamethasone in critically-ill COVID-19 patients was associated with ameliorated disease outcomes [2]. Furthermore, several randomised controlled trials have also corroborated the findings of the RECOVERY trial, demonstrating significantly improved outcomes in critically-ill COVID-19 patients treated with corticosteroids [3]. In the aftermath of these findings, the US National Institute of Health (NIH) vouches for the uptake of dexamethasone in patients with severe COVID-19 [4]. In the same vain, the WHO recommends the use of systemic corticosteroids in patients with severe manifestations of COVID-19 [5]. However, the WHO advises against the use of steroids in non-critically ill patients [5]. Nevertheless, there is paucity of data that evaluates the efficacy of a low-dose corticosteroid regimen in halting the progression of non-severe COVID-19.

Corticosteroids possess anti-inflammatory properties, which can be instrumental in modulating the aberrant, hyperactive immune response that remains a hallmark of COVID-19 [3]. These anti-inflammatory properties aim towards controlling the overdrive in cytokine production by inhibiting the transcription of certain pro-inflammatory cytokines that ultimately foment an esoteric cytokine storm [3]. Interestingly, recent literature has shown that glucocorticoids can indeed reduce inflammatory cell exudation and phagocytosis in the early stages of inflammation, thereby inhibiting excessive proliferation of fibroblasts that is associated with adverse disease outcomes [3].

The landmark RECOVERY trial was the first to demonstrate that the use of corticosteroids in critically-ill COVID-19 patients is indeed associated with reduced all-cause mortality and thus improved disease outcomes [2]. The trial evaluated patients who were hospitalized with a severe COVID-19 infection. Subjects were randomly assigned to either receive a 6 mg dose of once daily dexamethasone, or to receive the usual standard of care alone. The results from the trial divulged a reduced 28-day mortality rate in the dexamethasone group when compared to the non-steroid group (22.9% vs 25.7%, age-adjusted rate ratio = 0.83; 95% confidence interval = 0.75 to 0.93; P < 0.001) [2]. Furthermore, the 28-day mortality decreased from 41.3% to 29.3% in those receiving invasive mechanical ventilation, and from 26.2% to 23.3% in those receiving supplementary oxygen alone. Additional favourable outcomes within the dexamethasone group included shorter hospital stays and reduced progression to ventilation [2].

Contrarily, several studies have demonstrated that corticosteroid use in COVID-19 patients, while beneficial in terms of reducing overall mortality, might indeed be associated with reduced viral clearance and an increased risk of developing secondary infections [6]. Moreover, a number of studies have vouched against the use of corticosteroids in the early phases of COVID-19, citing their immunosuppressive effects, delaying of viral clearance, and numerous complications as evidenced by their anecdotal use in the middle eastern respiratory syndrome coronavirus (MERS-CoV) and severe acute respiratory syndrome coronavirus 1 (SARS-CoV-1) outbreaks [7]. In contrast, the early administration of low-dose corticosteroids for a short-term in non-severe COVID-19 infections has the potential to thwart the rapid progression of the disease course and ameliorate clinical outcomes while minimizing unnecessary harm [[8], [9], [10]]. To this date, there is scarcity of data regarding the efficacy and safety of low-dose corticosteroids for a short-term in the treatment of mild and moderate cases of COVID-19.

Almas et al. conducted a retrospective cohort study that evaluated data from 25 symptomatic patients who had a confirmed diagnosis of COVID-19 based on RT-PCR [11]. The patients followed an identical therapeutic regimen, comprising azithromycin 500 mg (OD), cetirizine 10 mg OD and acetaminophen 500 mg BD [11]. The study subjects were then randomised; 12 of the 25 patients were commenced on low-dose steroids (prednisolone 5 mg QDS) for a period of 7 days while the remaining 13 patients underwent standard treatment bereft of steroids [11]. The vast majority of participants of the study demonstrated an array of clinical signs, symptoms and findings, which were in concordance with the symptomatology of mild-to-moderate COVID-19. The baseline comorbidities of study participants were also taken into account and were managed using routine pharmacological regimens [11].

Pertinently, the mortality rate in the non-steroid group remained at a soaring 61.5% whereas the group receiving steroids had a significantly reduced mortality rate hovering at 8.3% (p = 0.005) [11]. Similarly, the presence of acute respiratory distress syndrome in the steroid group was significantly lower than that the non-steroid group (16.7% vs 84.6%, p = 0.002) [11]. Additionally, the risk of other adverse outcomes including shock, secondary infection and acute kidney injury was also lower in the steroid group [11]. Upon comparing relevant laboratory findings between the two groups, it was noted that by the end of the 7-day steroid course, the steroid group manifested palpably better results. These consisted of distinguishably lower D-Dimer levels (194.78 ng/mL vs 293.91 ng/mL, p = 0.001), LDH levels (201.41 U/L vs 242.19 U/L, p = 0.011), CRP levels (14.53 mg/dL vs 44.43 mg/dL, p < 0.001) and the total lymphocyte count (8.9103/μL vs 9.74 103/μL, p = 0.552) among the steroid group [11]. Finally, it was also observed that steroid therapy curtailed the length of hospital stay within the steroid group (14.23 vs 20.16 days, p < 0.001) [11].

Over the course of the pandemic, a myriad of other studies have also scrutinised the role and efficacy of corticosteroids in curbing the adverse outcomes elicited by COVID-19. A cohort study from Wuhan, China has revealed that among patients developing ARDS following COVID-19, methylprednisolone treatment is effective in curbing the mortality rate, with those receiving the aforementioned treatment demonstrating a 46.0% mortality rate in contrast to 61.8% in those who did not receive methylprednisolone [4]. Another study from Spain concluded that steroid treatment lowered mortality by 41.8% relative to treatment without steroids [12]. Similarly, in another study by Selvaraj et al. steroid therapy has boasted potential by eliciting a 77.98% fall in peak CRP levels after the commencement of dexamethasone [13]. A study comparing outcomes between patients receiving early steroids upon ICU submission (n = 485), patients receiving delayed steroids (n = 206), and patients never receiving steroids (n = 191) has also yielded noteworthy results [14]. It was noted that patients who received early steroids had a lower mortality rate (30.3%) compared to those who received a delayed steroid treatment (44.2%) and those who never received steroids (36.6%) [14]. Similarly, the 7-day mortality was also lower in the early steroid group (7.2% vs 15.2% in the never group) [14]. Secondary outcomes included more ventilator free days, shorter ICU stays, and a decrease in secondary infections [14].

The advent of the COVID-19 vaccine indubitably hones the potential to effectively end the pandemic. Over the next few months, a multitude of countries will begin mass vaccination of their vulnerable masses. It is well-said that the “light at the end of the tunnel” is indeed possible to envisage with the aforesaid advent; however, in resource-deprived nations that presently do not have the capacity to begin such mass vaccination, treatment with low-dose, short-course corticosteroids might be the way forward. It must be borne in mind that steroids could potentially prove to be a bane if a delicate equilibrium amongst their anti-inflammatory and immunosuppressive properties is not maintained. In this context, when—and indeed where—to draw the line with regards to their dosage, route, and mode of administration remains somewhat elusive. Nevertheless, their efficacy in thwarting the progression, even in instances of a non-severe COVID-19 infection, cannot be discounted.

## Ethical approval

NA.

## Source of funding

None.

## Author contribution

TA conceived the idea, wrote the first draft of the manuscript, and revised the manuscript critically.

SH and AH conducted the literature search and helped in revising the initial draft.

AH and ME helped with the statistical analysis of the original study quoted in the paper and also helped in evaluating the statistical significance of the results quoted from other studies.

AWK and AH helped in critically revising the finalised manuscript. All authors revised and approved the final version of the submitted manuscript.

## Trial registry number

1. Name of the registry: N/A
2. Unique Identifying number or registration ID: N/A
3. Hyperlink to your specific registration (must be publicly accessible and will be checked): N/A

## Guarantor

Talal Almas

RCSI University of Medicine and Health Sciences

123 St. Stephen's Green

Dublin 2, Ireland

Talalalmas.almas@gmail.com

+353834212442.

## Consent

N/A.

## Declaration of competing interest

None.

## References

[1] Zhu N., Zhang D., Wang W., Li X., Yang B., Song J., Zhao X., Huang B., Shi W., Lu R., Niu P., Zhan F., Ma X., Wang D., Xu W., Wu G., Gao G.F., Tan W. (2020). A novel coronavirus from patients with pneumonia in China, 2019. N. Engl. J. Med..

[2] Dexamethasone in Hospitalized Patients with Covid-19 (2020). Preliminary report. N. Engl. J. Med..

[3] Tomazini B.M., Maia I.S., Cavalcanti A.B. (2020). Effect of dexamethasone on days alive and ventilator-free in patients with moderate or severe acute respiratory distress syndrome and COVID-19: the CoDEX randomized clinical trial. J. Am. Med. Assoc..

[4] Centers for Disease Control Prevention Clinical care guidance for healthcare professionals about coronavirus (COVID-19). https://www.cdc.gov/coronavirus/2019-ncov/hcp/clinical-care.html.

[5] WHO (September 2020). Corticosterids for COVID-19: Living guidance. https://www.who.int/publications-detail-redirect/WHO-2019-nCoV-Corticosteroids-2020.1.

[6] Shuto H., Komiya K., Yamasue M., Uchida S., Ogura T., Mukae H., Tateda K., Hiramatsu K., Kadota J.-I. (2020). A systematic review of corticosteroid treatment for noncritically ill patients with COVID-19. Sci. Rep..

[7] Li Q., Li W., Jin Y., Xu W., Huang C., Li L., Huang Y., Fu Q., Chen L. (2020). Efficacy evaluation of early, low-dose, short-term corticosteroids in adults hospitalized with non-severe COVID-19 pneumonia: a retrospective cohort study. Infect. Dis. Ther..

[8] Russell C.D., Millar J.E., Baillie J.K. (2020). Clinical evidence does not support corticosteroid treatment for 2019-nCoV lung injury. Lancet.

[9] Fadel R., Morrison A.R., Vahia A., Smith Z.R., Chaudhry Z., Bhargava P., Miller J., Kenney R.M., Alangaden G., Ramesh M.S., Force H.F.C.-M.T. (2020). Early short-course corticosteroids in hospitalized patients with COVID-19. Clin. Infect. Dis..

[10] Fu H.Y., Luo Y., Gao J.P., Wang L., Li H.J., Li X., Xue L., Tang X.Q., Li H.W., Du Y.R. (2020). Effects of short-term low-dose glucocorticoids for patients with mild COVID-19. BioMed Res. Int..

[11] Almas T., Ehtesham M., Khan A., Khedro T., Hussain S., Kaneez M., Alsufyani R., Almubarak D., Alahmed F., Alaeddin H. (2020). Safety and efficacy of low-dose corticosteroids in patients with non-severe coronavirus disease 2019: a retrospective cohort study. Cureus.

[12] Fernández-Cruz A., Ruiz-Antorán B., Muñoz-Gómez A. (2020). A retrospective controlled cohort study of the impact of glucocorticoid treatment in SARS-CoV-2 infection mortality. Antimicrob. Agents Chemother..

[13] Selvaraj V., Dapaah-Afriyie K., Finn A., Flanigan T.P. (2013). Short-term dexamethasone in sars-CoV-2 patients. R. I. Med. J..

[14] Monedero P., Gea A., Castro P., Candela-Toha A.M., Hernández-Sanz M.L., Arruti E., Villar J., Ferrando C. (2021 Jan 4). COVID-19 Spanish ICU Network. Early corticosteroids are associated with lower mortality in critically ill patients with COVID-19: a cohort study. Crit. Care.

